# Sources of Lead in Cocoa and Chocolate

**Published:** 2006-05

**Authors:** William I. Manton

**Affiliations:** University of Texas, Dallas Richardson, Texas, E-mail: manton@udallas.edu

The article “Lead Contamination in Cocoa and Cocoa Products: Isotopic Evidence of Global Contamination” (Rankin et al. 2005) has attracted international attention (crienglish.com 2005) because of an interview that Rankin granted a reporter from *Science News* (Raloff 2005). Raloff’s report makes it generally known that Rankin et al.’s study was commissioned by the American Environmental Safety Institute (AESI).

I feel that the study by Rankin et al. contains careless and misleading science, specifically Figures 2 and 4, which purport to display the isotopic measurements they listed in Table 3. However, there appear to be twice as many symbols representing chocolate products in each of these figures as there are analyses in Table 3. On closer examination, it seems that the data are shown twice: first as given in Table 3, and then a second time with each data point shifted to the right and slightly down. Furthermore, in both figures “Cocoa powder 2” (Table 2) is incorrectly plotted. If the second set of points is omitted and the “Cocoa powder 2” is replotted, a different picture emerges, one in which the chocolate products and the cocoa define two trends that lie on either side of the trend of the aerosols. Thus, the statement by Rankin et al. (2005) that “the plot shows that isotopic compositions of all the chocolate products overlap with those of lead aerosols” is unsupported by their primary measurements, and all ensuing arguments pertaining to the contamination of chocolate products during manufacture are invalid.

There are other statements in their article that I consider irresponsible. For example, Rankin et al. (2005) stated that the presence of contaminant lead in cocoa bean shells is substantiated by the concentrations of lead in the soil profiles. Such a view ignores that lead is a naturally occurring element and that the lead content of a soil depends on how much the parent material contained and the manner in which the soil developed. In the United States, agricultural soils with the lowest lead contents are leached ultisols containing 8 ppm and those with the highest are the clay-rich vertisols with 17 ppm (Helmke 2000, Table 1.4). Seen in this light, the value of 14.2 ppm for the average of the Nigerian samples suggests minimally contaminated soil. If Rankin et al. (2005) had included the isotope ratios of the aerosols in their Figure 3, it would have been obvious that the soil leads occupied a different field. In Figure 2, for example, there are no aerosol leads with ^208^Pb/^207^Pb ratios > 2.48, whereas in Figure 3, 14 of 18 soil samples have ratios greater or equal to this value. The variability of the isotope ratios of the soils does not reflect the multiple sources of contamination that Rankin et al. (2005) invoked but rather the variable ratios of thorium to uranium in the old granitic rocks from which the soils were derived.

Another criticism I have is that Rankin et al. (2005) blurred the facts as they proceeded with their arguments. In “Sample collection and preparation” and in the title of Table 1, they pointed out that two types of beans and shells were analyzed, those taken directly from the husk (by which they presumably mean the pod) and those that had been fermented and dried. In Table 4, however, the distinction between the two types of shell was unspecified and they are listed as “Shell 1” and “Shell 2.” Thereafter, both shells are treated as the same entity, with high lead contents inferred to result from atmospheric contamination. But the shells of the beans taken directly from the pod should never have been exposed to atmospheric deposition. Why then do they have lead concentrations and isotope ratios similar to those that were fermented? The simplest explanation is that both were accidentally contaminated after they were collected.

Finally, having been involved in the case of AESI *v*. Mars, Inc. et al. (AESI 2002), I know that two of Rankin’s coauthors were expert witnesses for the plaintiffs and that at least one of them was compensated. I am surprised that they declared that they had no competing interests.
